# Integrated Mechanical and Cardiopulmonary Adaptations During Repeated Jumps in Volleyball Players: Insights from CPET Analysis

**DOI:** 10.3390/sports14010034

**Published:** 2026-01-08

**Authors:** Ștefan Adrian Martin, Isabella Pelaghie, George Mihăiță Gavra, Gabriela Szabo, Roxana Maria Martin-Hadmaș

**Affiliations:** 1 Department of Physiology, Center for Advanced Medical and Pharmaceutical Research, George Emil Palade University of Medicine, Pharmacy, Science and Technology of Târgu Mures, Gheorghe Marinescu 38, 540139 Târgu Mures, Romania; 2 Center for Advanced Medical and Pharmaceutical Research, George Emil Palade University of Medicine, Pharmacy, Science and Technology of Târgu Mures, Gheorghe Marinescu 38, 540139 Târgu Mures, Romania; 3 Department of Community Nutrition and Food Safety, George Emil Palade University of Medicine, Pharmacy, Science and Technology of Târgu Mures, Gheorghe Marinescu 38, 540139 Târgu Mures, Romania

**Keywords:** volleyball performance, cardiopulmonary exercise testing, countermovement jump, oxygen kinetics, neuromuscular fatigue

## Abstract

Volleyball physical performance relies on the interaction between mechanical power, metabolic efficiency, and ventilatory regulation during repeated high-intensity actions. This study examined mechanical and cardiopulmonary responses during three consecutive 15 s countermovement jump bouts in female volleyball players, using simultaneous cardiopulmonary exercise testing. Eighteen female athletes (18–28 years) completed the protocol with 60 s active recovery between efforts. Mechanical performance showed a progressive decline (*p* < 0.01), with jump height decreasing from 20.59 ± 3.04 cm to 19.30 ± 3.23 cm and power output from 15.80 ± 2.61 to 14.83 ± 2.25 W/kg (*p* = 0.001). Oxygen uptake (VO_2_) increased from 16.40 ± 6.73 to 20.87 ± 6.08 mL/min/kg (*p* = 0.002), while respiratory exchange ratio (RER) rose above 1.0, suggesting a growing anaerobic contribution. VE/VO_2_ and PetO_2_ also increased significantly (*p* < 0.001), indicating ventilatory adjustment to metabolic stress. Despite these adaptations, recovery between efforts appeared incomplete, reflected by persistent ventilatory and metabolic activation. These findings suggest moderate oxidative efficiency and partial fatigue compensation under short recovery conditions. The testing model may serve as a practical approach to evaluate the interplay between mechanical and metabolic performance and to refine individualized conditioning strategies in volleyball players.

## 1. Introduction

Across all sports, training methodologies are continuously refined to enhance functional adaptation and optimize physical performance. Athletes develop and refine strength, speed, endurance, coordination, and mobility, while coaches adjust training plans according to individual and collective data [1]. In this process, sport training integrates both general and sport-specific components, each essential for optimizing physical and functional capacity [2,3,4]. Volleyball, in particular, has undergone substantial evolution in match intensity, tactical, and overall physical and biomechanical requirements [5,6]. These changes reflect advancements in both training and research methodologies [7], which have collectively contributed to improved athletic performance.

Several papers have emphasized that an insufficient level of general physical preparation during childhood and adolescence—often overshadowed by early technical and tactical specialization—may negatively affect later physical performance [8]. Deficiencies in the force–velocity profile, inter- and intramuscular coordination, and tendon–muscle stiffness can persist into adulthood, ultimately limiting an athlete’s ability to further improve. This is the main reason why authors suggest that training should align with the mechanical demands of volleyball [9] while remaining specific to each age group. Further on, papers suggest that training periodization should accurately reflect both the mechanical and metabolic demands of volleyball, with particular emphasis on the high-frequency jumping and landing actions that define the sport. Yet, when examining many current training practices and their outcomes, it appears that some general principles of physical preparation—and consequently, the capacity to sustain and adapt to repeated high-intensity efforts—have been overlooked. This highlights the need to reintegrate the study of cardiopulmonary (CP) adaptation within the specific context of volleyball.

Volleyball-specific training, characterized by frequent jump–landing, rapid directional changes, and explosive efforts, requires not only osteogenic and neuromuscular adaptations—such as improved bone metabolism, enhanced mechanical stiffness, and better control during explosive actions—but also substantial metabolic, cardiac, and cardiopulmonary adjustments [10,11,12]. These include enhanced aerobic–anaerobic interaction, greater efficiency of the phosphagen and glycolytic systems, and faster ventilatory and oxidative responses during high-intensity intermittent efforts. The magnitude of these adaptations is influenced by factors such as hormonal status, nutritional intake (including calcium and vitamin D), overall training orientation, and load management [13,14].

Previous studies have shown that volleyball training methodologies have evolved in response to the increasing physical and tactical demands of the game [15,16,17,18]. While modern training approaches have become progressively more specialized at the senior level, the expression of sport-specific potential remains fundamentally constrained by the athlete’s general physical development achieved during the early stages of training. Consequently, training methods aimed at improving jumping and landing mechanics, as well as rapid movement execution, are frequently emphasized, as they enhance mobility and reactivity during match play [19]. Despite these advancements, relatively few studies [20] have implemented cardiopulmonary exercise testing (CPET) to assess performance capacity through volleyball-specific movement patterns while simultaneously examining metabolic efficiency, neuromuscular function, and game-specific performance outcomes [21,22,23,24,25]. Evidence indicates that the ability to sustain high-intensity intermittent efforts depends not only on anaerobic power [26] but also on aerobic capacity, which supports recovery between bouts of effort [27,28]. This interplay between energy systems determines the efficiency of repeated jumps, rapid transitions, and prolonged rallies—key determinants of match performance

Given that repeated jump sequences are central to volleyball, evaluating oxygen uptake kinetics, ventilatory responses, and recovery dynamics may provide valuable insights into energy system interaction and fatigue management, as previously reported in CPET-based studies [29]. Therefore, the present study aimed (i) to characterize the cardiopulmonary responses to a repeated CMJ protocol in female volleyball players and (ii) to examine their relationship with mechanical jump performance (height, power and contact time) across successive repetitions. In an exploratory manner, we also considered whether specific response patterns might have the potential, in future work, to contribute to the differentiation of athletes according to performance characteristics.

## 2. Materials and Methods

This cross-sectional study employed a volleyball-specific testing protocol performed concurrently with cardiopulmonary exercise testing (CPET) to examine oxygen kinetics and related performance changes across successive 15 s countermovement jump (CMJ) repetitions, in line with the study hypothesis. All testing procedures complied with the ethical standards of the Declaration of Helsinki and were approved by the University Ethics Committee (approval no. 3332 from 19 August 2024).

### 2.1. Study Overview

The study was conducted over a period of four months, between February and May 2025, in the Functional Explorations Laboratory of the Advanced Medical and Pharmaceutical Research Center, part of the “George Emil Palade” University of Medicine, Pharmacy, Science and Technology in Târgu Mureș, Romania. The first 30 days were allocated for participant recruitment, carried out through a public call for applications. This period allowed sufficient time for disseminating the announcement within local and national volleyball clubs and university sports associations, as well as for verifying eligibility criteria and obtaining medical clearance prior to enrollment.

The study sample initially consisted of 18 female volleyball players aged between 18 and 28 years. All participants were active players, taking part in official regional and/or national championships during the study period. Based on team records and self-report, all athletes had a minimum of 3 consecutive years of structured volleyball training (with most reporting between 5 and 10 years of organized practice) and were engaged in regular in-season training. In the 90 days preceding testing, players followed a broadly comparable training schedule, typically involving approximately 3–5 on-court volleyball sessions and 1–2 strength and conditioning sessions per week, corresponding to an overall weekly training volume of about 8–12 h.

Data collection was carried out during the competitive season and required access to the cardiopulmonary exercise testing (CPET) system, medical screening and staff supervision within limited time windows. Under these constraints, the sample size was determined by in-season player availability and the practical feasibility of CPET, rather than by a predefined statistical target. All eligible players who responded to the open announcement, were medically cleared, and were available and willing to complete the CPET protocol during the testing period were included, resulting in 18 participants. No formal a priori sample-size calculation was performed, and the present study should therefore be considered exploratory. The inclusion of female athletes within the 18–28-year age range was justified by the need to ensure a homogeneous group in terms of biological maturity, hormonal stability and training background. This range corresponds to the period of peak physical performance and competitive readiness in volleyball, thereby minimising variability related to growth or age-associated decline. The decision to include only female athletes was also influenced by the absence of organised male volleyball teams with a comparable competitive structure in the geographical area where the research was conducted, which prevented the formation of a representative male cohort.

To ensure a consistent level of competitiveness and recent exposure to match play, all participants were required to have completed at least 90 consecutive days of structured training, including participation in official competitions, prior to testing. Athletes playing the libero position were excluded from the sample, given the lower relevance of repeated vertical jumping actions to their specific positional demands. Furthermore, all participants had to be clinically healthy and free from any acute or chronic respiratory, cardiac, endocrine or metabolic conditions that could limit exercise testing or interfere with data collection and interpretation in relation to the study objectives and hypotheses. Failure to meet these inclusion criteria resulted in non-acceptance, while any condition acquired during the study period led to exclusion from the research program. Written informed consent to participate was mandatory for all subjects prior to enrollment.

#### Testing Procedures and Measurements

The athletes reported individually for evaluation, following the order of recruitment. In total, each participant visited the laboratory three times (*n* = 3). During the first visit, further referred as V1, assessments were conducted under basal conditions and included a complete anthropometric and basic clinical evaluation. The clinical screening aimed to confirm the absence of any acute or chronic conditions and consisted of: a resting electrocardiogram (ECG) performed using a MAC 5500^®^ 12-lead electrocardiograph (GE Healthcare, Chicago, IL, USA); an ultrasound examination using a Vivid E9 BT12^®^ echocardiograph (GE Healthcare, Chicago, IL, USA); and a general health assessment conducted with diagnostic equipment available at the Center for Advanced Medical and Pharmaceutical Research (CCAMF), including an ergospirometry system (Cortex Metalyzer 3B^®^) (Cortex Biophysik, Leipzig, Germany). During the same visit, the anthropometric measurements were performed using Tanita MC-780 MA (Tanita Corp., Tokyo, Japan) analyzer for body composition, and Seca 213 (Seca GmbH & Co. KG, Hamburg, Germany) for height. Menstrual cycle information was collected during pre-testing screening. All participants reported regular menstrual cycles and none were using hormonal contraceptives. However, evaluations were not synchronized with a specific menstrual cycle phase, and testing occurred according to each athlete’s availability. Recent training load was partially controlled through pre-test instructions, requiring participants to avoid strenuous exercise in the 24 h preceding laboratory testing. All anthropometric data were collected during this visit and included the following parameters: body height (m), body weight (kg), active muscle mass (kg), fat mass (%), and segmental body composition of the upper and lower limbs (right and left), assessed separately for adipose and active muscular tissue distribution.

The second laboratory visit, further referred as V2 was scheduled two days later and consisted of a single CMJ test. Prior to the testing, all participants completed a volleyball-specific warm-up lasting approximately 15 min, which included 5 min of general aerobic activation (light jogging and joint mobility exercises), 5 min of dynamic stretching (hips, knees, and ankles), and 5 min of sport-specific drills, including submaximal vertical jumps and short accelerations, to ensure neuromuscular readiness for explosive efforts. After completing the warm-up, participants performed the CMJ test three times successively, with a one-minute rest interval between attempts. The highest recorded value among the three trials was retained as the reference performance for subsequent comparisons. Following this evaluation, a simulation and familiarization session with the complete testing protocol was conducted to ensure proper understanding of the procedure and consistency in execution during the final assessment (V3).

**Figure 1:**
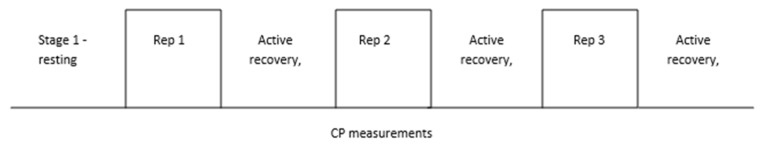
Structure of the volleyball-specific testing protocol including CMJ and recovery phases.

Two days later, the athletes attended the third and final visit, further referred to as V3, which corresponded to the implementation of the full testing protocol. Following the same warm-up procedure, participants underwent the functional exercise assessment, which included seven consecutive stages as follows: Stage 1—rest monitoring (baseline cardiopulmonary recording), Stage 2—repetition 1 (rep 1), 15 s CMJ with hands on hips, Stage 3—active recovery 60 s, Stage 4—repetition 2, 15 s CMJ with hands on hips, Stage 5—active recovery 60 s, Stage 6—repetition 3, 15 s CMJ with hands on hips, and Stage 7—active recovery 60 s, as illustrated in Figure 1.

### 2.2. Measurements During the 15 s CMJ Test

#### 2.2.1. Dynamic Strength

Mechanical performance during the single and 15 s CMJ tests was assessed using the Optojump system (Microgate, Bolzano, Italy). A predefined measurement protocol was followed, and the following parameters were recorded for each jump: center of gravity height (centimeters, cm), contact time (milliseconds, ms), and power (Watt per kilogram, w/kg). During testing, participants received active verbal encouragement to maintain a maximal effort throughout the 15 s interval. The data analysis was conducted by calculating the mean values over the entire 15 s CMJ as well as by segmenting the working time into three phases, as follows: Phase 1 corresponding to the 0–5 s interval, Phase 2 to the 5–10 s interval, and Phase 3 to the 10–15 s interval of the 15 s effort. The 60 s recovery period was subdivided into three consecutive 20 s intervals for descriptive analysis. This segmentation was selected for methodological convenience, allowing temporal resolution of recovery dynamics while reducing breath-by-breath variability inherent to explosive tasks. No specific physiological thresholds were assumed for these intervals.

#### 2.2.2. Integration of Cardiopulmonary (CP) Measurements During the CMJ Protocol

CP measurements were performed continuously using the Cortex Metalyzer 3B R3 system. The equipment was calibrated before each testing using a reference gas mixture (15% O_2_ and 5% CO_2_) while the flow sensor was calibrated with a 3 L syringe to ensure accuracy of ventilation measurements. Ambient conditions (temperature, barometric pressure, and relative humidity) were recorded automatically and integrated into the software for correction of gas volume and flow.

During the protocol, we measured breath-by-breath the following parameters: oxygen uptake (VO_2_), carbon dioxide output (VCO_2_), and respiratory exchange ratio (RER). Additionally, ventilatory equivalents for oxygen (VE/VO_2_) and carbon dioxide (VE/VCO_2_), as well as end-tidal partial pressures of oxygen (PETO_2_) and carbon dioxide (PETCO_2_), were continuously recorded Gas exchange was measured breath-by-breath exported with a 3 s moving average. For the intermittent jumping protocol, each work interval lasted 15–17 s and was followed by an active-recovery period. For every work and recovery interval we calculated the mean VO_2_, VCO_2_, VE and respiratory exchange ratio (RER) over the entire 15–20 s duration. Because of the very short and intermittent nature of this protocol, these 15–20 s averaged values are used as descriptive markers of the acute cardiopulmonary response and recovery, rather than as classical steady-state CPET outcomes. Participants wore a low-resistance facemask connected to the turbine flowmeter and gas analyzer, ensuring minimal leakage and comfort during jumping and recovery phases. The Cortex Metalyzer 3B system was interfaced with the Metasoft Studio software, version 5.10.0, which allowed continuous visualization and synchronization of ventilatory and performance parameters throughout all 7 stages of the protocol.

#### 2.2.3. Statistical Analysis

All data were checked for completeness and accuracy prior to analysis. Descriptive statistics were expressed as mean ± standard deviation (SD) for continuous variables and as frequency (*n*, %) for categorical variables. The Shapiro–Wilk test was applied to assess the normality of distributions for all continuous variables.

Group comparisons across repetitions and within the three time segments of each 15 s CMJ were performed using repeated-measures analysis of variance (RM-ANOVA). Assumptions of normality and sphericity were examined for each model; when Mauchly’s test indicated sphericity violations, Greenhouse–Geisser corrections were applied. For variables in which distributional assumptions were not met, non-parametric alternatives (Friedman test with Wilcoxon signed-rank post hoc comparisons) were additionally reported to support the robustness of the findings. Pairwise contrasts were adjusted using Bonferroni correction.

Effect sizes were reported as partial eta-squared (ηp^2^) for repeated-measures effects and Cohen’s d for pairwise comparisons, in line with current recommendations for interpreting practical relevance in small-sample exploratory designs. A post hoc sensitivity assessment indicated that the observed effect sizes (ηp^2^ range 0.18–0.42 for major outcomes such as VO_2_, VCO_2_, jump height, and power output) correspond to an achieved statistical power above 0.80 for these primary variables, supporting the stability of the main inferential results despite the limited sample.

Relationships between CPET-derived parameters and mechanical indices were examined using Pearson’s or Spearman’s correlation coefficients according to data distribution. Statistical significance was set at *p* < 0.05 for all analyses. All analyses and graphical representations were performed using GraphPad Prism version 9.0 (GraphPad Software, San Diego, CA, USA).

## 3. Results

### 3.1. General Characteristics and Descriptive Data (V1–V2)

Participants had a mean height of 1.80 ± 0.07 m and a body weight of 68.53 ± 7.86 kg, with an average body fat percentage of 22.51 ± 4.65% and a skeletal muscle mass of 50.16 ± 3.74 kg. During V2, the single CMJ test performed under baseline conditions—used as a reference for subsequent repetitions—recorded a mean jump height of 25.06 ± 3.04 cm, ranging from 20.20 to 31.30 cm (min to max).

### 3.2. Mechanical Performance During the 15 s CMJ Test

During the first repetition, contact time was 0.71 ± 0.13 s, with a corresponding flight time of 0.40 ± 0.03 s, resulting in a jump height of 20.59 ± 3.04 cm, equivalent to 15.80 ± 2.61 W/kg of power. The jump height recorded in the first repetition represented 82.16% of the reference value (25.06 cm), corresponding to a 17.84% decrease compared to the baseline performance. During the second repetition, the mean jump height was 20.57 ± 3.65 cm, with a contact time of 0.74 ± 0.15 s and a power output of 15.59 ± 2.52 W/kg. The results showed a 17.92% decrease from the reference value and a negligible −0.08% variation compared with the first repetition. During the third repetition, the contact time was 0.73 ± 0.11 s, the jump height 19.30 ± 3.23 cm, and the power output 14.83 ± 2.25 W/kg. Compared with the reference value, the third repetition exhibited a 22.99% reduction in jump height, confirming a progressive decline in mechanical performance across repetitions.

### 3.3. Comparative Analysis of the Three CMJ Repetitions (V3) Across Successive Time Phases (0–5 s, 5–10 s, 10–15 s)

Table 1 presents the mean ± SD values of mechanical performance parameters recorded during the 0–5 s interval (Phase 1, according to the methodological segmentation of the 15 s CMJ effort) for the three repetitions (Rep 1–3). The corresponding repeated-measures ANOVA results, including *p* values, *F* statistics, and *R*^2^ coefficients, are further detailed.

The results indicate relatively stable mean values across repetitions, with small variations in jump height, power, and contact time that did not reach statistical significance sin 0–5 s’ phase. Similar analyses were performed for the subsequent time, as further approached through Table 2 and Table 3.

During the 5–10 s interval, mean values of mechanical performance variables showed no significant differences across repetitions. Data corresponding to the final 10–15 s interval are summarized in Table 3.

During the final 10–15 s phase, statistically significant differences were observed in jump height (*p* = 0.006) and power output (*p* = 0.012) across repetitions, whereas contact time showed no significant difference (*p* = 0.437). These results conclude the mechanical performance analysis across the three-time intervals.

### 3.4. CP Responses During the 15 s CMJ Tests

Across all three repetitions, a gradual increase in oxygen uptake was observed, with mean values rising from 1.09 ± 0.41 L/min (16.40 ± 6.73 mL/min/kg) in the ***first*** repetition, to 1.30 ± 0.40 L/min (19.29 ± 6.43 mL/min/kg) in the ***second*** repetition, and 1.41 ± 0.39 L/min (20.87 ± 6.08 mL/min/kg) in the ***third***. Similarly, VCO_2_ increased from 0.86 ± 0.34 L/min during the first repetition, to 1.42 ± 0.45 L/min and 1.59 ± 0.45 L/min in the subsequent repetitions. The respiratory exchange ratio (RER) reflected a corresponding evolution, shifting from 0.79 ± 0.07 in the first repetition to 1.09 ± 0.13 and 1.11 ± 0.14 in the second and third, respectively. These dynamics were accompanied by stable ventilatory equivalents (VE/VO_2_, VE/VCO_2_) across repetitions, as it is detailed in the following Table 4, Table 5 and Table 6.

During this initial phase of the 15 s CMJ, VO_2_ increased markedly between repetitions (*p* = 0.0001), with parallel differences noted for VO_2_/HR (*p* = 0.0008) and VE/VO_2_ (*p* = 0.0001). PetO_2_ and PetCO_2_ were significantly different (*p* = 0.0297 and *p* = 0.0006, respectively), indicating consistent ventilatory adjustments during the early phase of effort. In contrast, VE/VCO_2_ remained relatively stable (*p* > 0.05).

During the second phase of the 15 s CMJ, significant differences were observed for VO_2_ (*p* = 0.002), indicating progressive increases across repetitions. End-tidal gas parameters also showed significant variation, with both PetO_2_ (*p* = 0.0004) and PetCO_2_ (*p* = 0.0076) demonstrating consistent upward trends. In contrast, VO_2_/HR (*p* = 0.231) and VE/VCO_2_ (*p* = 0.1789) remained relatively stable across repetitions, as further described in Table 5.

During the final phase, statistically significant differences were found for VO_2_ (*p* = 0.0014) and VE/VO_2_ (*p* = 0.0001), indicating a continued rise in oxygen uptake. End-tidal pressures also exhibited consistent variation, with significant changes in both PetO_2_ (*p* = 0.0001) and PetCO_2_ (*p* = 0.0160). VO_2_/HR failed to change (*p* = 0.0552), while VE/VCO_2_ remained stable across repetitions (*p* = 0.1718), as further described in Table 6.

### 3.5. Cardiopulmonary Recovery Dynamics Between Repetitions

During the first recovery period (Stage 3), VO_2_ was 22.63 ± 4.20 mL/min/kg, whereas during the second recovery period (Stage 5) it increased to 23.48 ± 4.04 mL/min/kg. Ventilatory equivalents also presented higher mean values in the second recovery, with VE/VO_2_ = 26.60 ± 3.57 L/min compared to 24.77 ± 3.95 L/min, and VE/VCO_2_ = 24.81 ± 2.34 L/min compared to 24.84 ± 2.12 L/min. PetO_2_ increased from 100.9 ± 6.45 mmHg to 104.1 ± 5.55 mmHg, while PETCO_2_ remained relatively stable (42.11 ± 3.39 mmHg vs. 42.23 ± 4.23 mmHg) across the two recovery periods. VCO_2_ also rose from 1.52 ± 0.23 L/min to 1.69 ± 0.24 L/min, accompanied by a higher respiratory exchange ratio (RER = 1.07 ± 0.08 vs. 0.99 ± 0.11), indicating a greater ventilatory contribution during the second recovery stage.

Statistical analysis confirmed significant differences for VE/VO_2_ (*p* = 0.0001, r = 0.321, 95% CI = −2.80 to −1.12), VCO_2_ (*p* = 0.0001, r = 0.611, 95% CI = −0.275 to −0.170), and PETO_2_ (*p* = 0.0001, r = 0.472, 95% CI = −4.50 to −2.37).

Conversely, VO_2_, VO_2_/HR, VE/VCO_2_, and PetO_2_ did not show overall significant differences between the two recovery periods (*p* > 0.05). However, to better describe the dynamics of recovery, the 60 s interval was divided into three consecutive 20 s stages. When analyzed in detail, VO_2_ was significantly different between recovery periods during the first (*p* = 0.0339, r = 0.266, 95% CI: −2.81 to −0.12) and second 20 s stages (*p* = 0.0204, r = 0.309, 95% CI = −2.59 to −0.25), while the third stage did not reach statistical significance (*p* = 0.1022, r = 0.168, 95% CI = −2.08 to 0.20).

**Figure 2:**
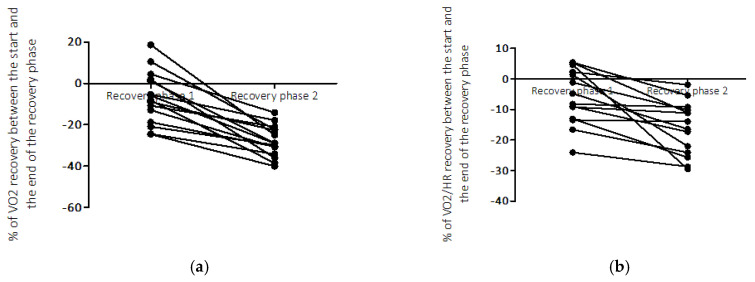

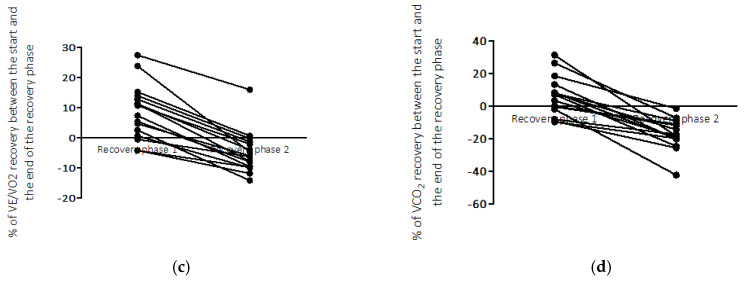
Percentage change in cardiopulmonary recovery parameters—VO_2_ (**a**), VO_2_/HR (**b**), VE/VO_2_ (**c**), VCO_2_ (**d**)—showing the relative (%) progression from the start to the end of each 60 s recovery interval and the comparison between Recovery 1 and Recovery 2.

VE/VO_2_ showed a highly significant difference during the first 20 s of recovery (*p* = 0.0001, r = 0.834, 95% CI: −5.05 to −3.06), whereas no significant differences were observed during the subsequent 20–40 s and 40–60 s intervals (*p* > 0.05). For carbon dioxide output (VCO_2_), significant differences were consistently observed across all recovery stages: first 20 s (*p* = 0.0001, Mann–Whitney U = 25.50), second 20 s (*p* = 0.0006, r = 0.552, 95% CI = −0.275 to −0.09), and third 20 s (*p* = 0.0008, r = 0.540, 95% CI: −0.219 to −0.07). PETO_2_ also showed consistent variation across recovery time points, with significant differences in the first (*p* = 0.0001, r = 0.860, 95% CI = −8.05 to −5.13), second (*p* = 0.0210, r = 0.307, 95% CI = −3.91 to −0.37), and third recovery stage (*p* = 0.0363, r = −0.158, 95% CI = −3.04 to −0.11), as further detailed in Figure 2a–d.

## 4. Discussion

We investigated CP and mechanical performance during repeated CMJ in female volleyball players. The results showed a progressive decline in jump performance across repetitions, associated with increased oxygen uptake and ventilatory demand, as well as incomplete normalization of cardiopulmonary variables within 60 s. These findings indicate a progressive physiological strain across repetitions, reflected by increases in RER and VCO_2_. While these responses reflect substantial metabolic demand, interpretations regarding underlying increased metabolic strain or ventilatory compensation should be made cautiously, given the nature of the protocol.

### 4.1. Mechanical Performance and Neuromuscular Fatigue

The mechanical performance during repeated CMJ revealed a consistent and progressive decline across the three successive repetitions, with jump height decreasing from 20.59 ± 3.04 cm to 19.30 ± 3.23 cm, corresponding to a 22.99% reduction compared to the CMJ reference (25.06 cm). The associated decline in power output, from 15.80 ± 2.61 to 14.83 ± 2.25 W/kg is consistent with the development of fatigue-related performance decrements of neuromuscular fatigue despite short effort duration and brief inter-repetition recovery. This attenuation pattern is in line with other findings, which demonstrated that repeated jump or sprint protocols induce a rapid decline in explosive output [30] when the recovery interval is insufficient for full physiological restoration phosphocreatine resynthesis or pH restoration [31]. Nevertheless, it should be taken into account that our recovery time ratio was 4:1. More importantly, the magnitude of performance decline may reflect the interaction between protocol-induced fatigue and the athletes’ current conditioning level. This is consistent with literature showing that repeated efforts often reveal differences in neuromuscular conditioning. Thus, the jump values observed here likely reflect a genuine interaction between protocol-induced fatigue and the athletes’ actual training status. From a practical standpoint, supports the notion that repeated-jump assessments can offer insight into volleyball-specific readiness. These observations may help inform future training considerations.

In our study, the stability of contact time across repetitions suggests that participants maintained a consistent execution strategy despite accumulating fatigue, which aligns with previous findings showing that trained volleyball players tend to preserve technical stability even under metabolic stress [32]. However, considering that the applied recovery ratio was 4:1, one might expect a greater degree of performance and, consequently, better maintenance of jump efficiency within the group. The observed reduction in jump height and power therefore indicates that this recovery interval was insufficient for full physiological restoration. As reported by others [33], such declines likely reflect changes in motor output commonly reported during repeated explosive tasks toward the recruitment of slower motor units, alterations in force production characteristics, and reduced efficiency of the stretch–shortening cycle within the stretch–shortening cycle [34].

The degree of mechanical decline in our sample (23%) is within the range observed in repeated CMJ protocols involving competitive volleyball players (15–25%) [35], though the magnitude of RER and VO_2_ changes suggests differences in recovery-related cardiopulmonary responses. Nevertheless, the mechanical performance observed is lower than that typically reported. This may indicate that our athletes, while technically proficient, exhibit a less developed tolerance to repeated high-intensity efforts and therefore demonstrate lower overall performance as to the literature report [36,37].

### 4.2. Cardiopulmonary Dynamics and Oxygen Kinetics

The cardiopulmonary responses followed a progressive trend, with VO_2_ increasing from 16.40 ± 6.73 mL/min/kg in the first repetition to 20.87 ± 6.08 mL/min/kg in the third, accompanied by a marked rise in VCO_2_ (from 0.86, to 1.59 L/min) and RER (from 0.79 to 1.11). These data show a progressive rise in VO_2_ and VCO_2_ across repetitions, which is compatible with increasing physiological demand during repeated short-duration efforts. This physiological pattern is consistent with the VO_2_ slow component phenomenon observed during repeated high-intensity or intermittent exercise [38], in which oxygen uptake continues to rise over time. However, because we did not assess muscle fibre composition, twitch properties or local muscle metabolism, we cannot attribute these responses to specific mechanisms such as preferential type II fibre recruitment or reduced contractile efficiency, as seen in other papers [39]. Such explanations should be regarded as plausible interpretations rather than demonstrated causes. However, in our study, the metabolic response pattern suggests a transient but incomplete physiological adjustment between efforts, characterized by a steady rise in VO_2_ and VCO_2_ across repetitions without full restoration of energetic balance between efforts. This may suggest that ventilation increased proportionally to the rising metabolic demand, although the specific mechanisms cannot be determined from the current protocol, consistent with a growing contribution of non-oxidative metabolism, as suggested by RER > 1, though direct biochemical confirmation was not available Such a response pattern may define may reflect a transitional response pattern, where the metabolic system is capable of sustaining repeated high-intensity efforts but at the cost of progressive acidosis and reduced mechanical efficiency. Yet, previous published studies have shown that phosphocreatine resynthesis and reoxygenation of active musculature require 60–120 s depending on aerobic conditioning [40]. Consequently, the incomplete recovery observed here implies moderate oxidative efficiency within the studied group, information which provides a practical perspective on the athletes’ level of training.

In our sample, a notable observation is the divergent evolution of ventilatory equivalents: VE/VO_2_ increased from 24.77 ± 3.95 to 26.60 ± 3.57 L/min, while VE/VCO_2_ remained stable, indicating that ventilation scaled mainly to oxygen demand rather than to CO_2_ elimination. This suggests that ventilation increased in parallel with oxygen demand. However, variables such as VE/VO_2_ and end-tidal gases should be interpreted cautiously, given the movement-related variability inherent to jumping. The increase in VO_2_ and RER is compatible with higher overall metabolic strain, but the specific muscle-level mechanisms cannot be confirmed in the absence of direct measurements. The accompanying rise in PetO_2_ (from 100.9 to 104.1 mmHg) with stable PetCO_2_ (from 42.11 to 42.23 mmHg) reflects effective alveolar gas exchange without excessive ventilatory drift. Similar findings were reported by Martin B. et al. [41], who observed stable PetCO_2_ values during intermittent sprinting despite rising RER. Nevertheless, without direct measurements of local muscle oxygenation or metabolism (e.g., NIRS) we can only describe the integrated cardiopulmonary response and cannot disentangle central from peripheral contributions or confirm specific mechanisms such as heightened glycolytic fibre recruitment or reduced mechanical efficiency. Therefore, from a theoretical perspective, the increase in VO_2_ and RER can be attributed to heightened recruitment of glycolytic fibers, increased oxygen cost of ventilation, and reduced mechanical efficiency due to fatigue. According to Phillips D.B. et al. [42] as the muscle’s ability to utilize oxygen declines, ventilation compensates to maintain acid-base homeostasis, which explains the maintained VE/VCO_2_ ratio. This result further proves that CPET measurement during explosive tasks can capture subtle metabolic adjustments that are otherwise undetectable by mechanical measures alone.

### 4.3. High-Intensity Exercise-Induced Stress and Ventilatory Compensation

The increase in RER above 1.0 during the second and third repetitions provides evidence of metabolic disturbance resulting from accumulating anaerobic metabolism. The concomitant rise in VCO_2_ and VE/VO_2_ s commonly observed in high-intensity exercise and may reflect increased metabolic strain. Nonetheless, RER > 1 during intermittent recovery is expected and does not directly quantify acid–base status [43]. This pattern mirrors the ventilatory responses reported in supramaximal and intermittent protocols [44], where peripheral chemoreceptor stimulation drives an accelerated ventilatory response to counteract metabolic acidosis. However, unlike in continuous high-intensity exercise, the stable VE/VCO_2_ values observed in our study suggest that alveolar ventilation remained proportionally efficient throughout the repeated efforts. This balance indicates that participants increased minute ventilation in direct proportion to metabolic acidosis, without excessive hyperventilation—a sign of adequate ventilatory control under stress. Such regulation implies a functional synchronization between central respiratory command and peripheral feedback mechanisms, consistent with adaptive profiles described in trained populations [45,46].

Nonetheless, the persistence of high RER and elevated VCO_2_ values, despite the 4:1 recovery ratio, suggests that cardiopulmonary variables did not fully return toward baseline within 60 s. However, no direct measurements of phosphocreatine or acid–base balance were collected, so these interpretations remain indirect [47]. From this perspective, the observed ventilatory response represents a compensatory adaptation rather than a recovery-driven one, highlighting a state of functional resilience under metabolic constraint. Such findings suggest that even though the respiratory system efficiently regulated CO_2_ clearance, the underlying energetic restoration lagged, exposing an adaptive imbalance between ventilation and oxidative recovery.

### 4.4. Specific Recovery Profile and Training Implications

The 60 s active recovery between repetitions provided valuable insight into recovery kinetics. Significant differences were observed in VO_2_ and VE/VO_2_ during the first 40 s, after which values plateaued, suggesting that cardiopulmonary variables did not fully return toward baseline as early quoted. The early recovery phase showed the largest deviations (VO_2_, *p* = 0.0339; VE/VO_2_, *p* = 0.0001), confirming that both oxygen uptake and ventilatory demand remained elevated. Similar results were reported in intermittent-exercise protocols, where oxygen reuptake and CO_2_ clearance follow a biphasic pattern—a rapid initial component followed by a slower, incomplete phase [48]. However, in our study, the persistence of elevated VO_2_ and RER values throughout recovery may indicate differences in aerobic contribution to recovery within the cohort, although these interpretations should be considered cautiously. Volleyball players, though accustomed to high-intensity bursts, often display underdeveloped oxidative support due to the sport’s predominantly anaerobic character. Consequently, even small deficits in aerobic efficiency may influence repeated-exercise tolerance and delay full recovery between rallies or sets.

From a practical standpoint, the parallel decrease in jump performance from our sample and sustained ventilatory strain indicates that neuromuscular and cardiopulmonary trends appeared to evolve in parallel, suggesting a possible interaction between mechanical output and recovery dynamics. Further research incorporating biochemical or muscle oxygenation markers is needed to clarify this relationship [49]. These findings support the inclusion of aerobic–anaerobic transition training and interval recovery optimization in volleyball conditioning programs. By monitoring CPET parameters during sport-specific movements, professionals can individualize recovery intervals and training intensity, thereby improving oxygen kinetics, buffering capacity, and overall resilience to repeated various physical activities.

### 4.5. Functional and Methodological Perspectives

This study provides a different methodological framework by combining CMJ-based mechanical assessment with continuous CPET monitoring, allowing simultaneous evaluation of neuromuscular and metabolic adaptation. Unlike traditional jump tests that assess mechanical performance, this approach provides complementary information on cardiopulmonary–mechanical interactions. The observed relationships between jump decay, ventilatory compensation, and incomplete oxygen recovery may offer complementary insight into the interaction between mechanical and cardiopulmonary responses during explosive efforts. The protocol’s non-invasive and integrative nature suggests potential applicability for future functional assessments, pending further validation.

Future research should compare responses across different training levels and between sexes, and incorporate additional mechanistic tools—such as electromyography (EMG), near-infrared spectroscopy (NIRS), and muscle oxygen saturation (SmO_2_)—to validate local oxygen kinetics during explosive activity. The present sample of 18 participants is consistent with controlled, laboratory-based sport science protocols, yet it limits generalizability; results should therefore be interpreted with caution, particularly in light of the heterogeneous training status of the athletes.

Additionally, cardiopulmonary data were obtained during brief (15–20 s) intermittent efforts, which do not fully correspond to traditional continuous CPET. As such, VO_2_, ventilatory equivalents, and end-tidal parameters should be viewed as descriptive indicators of transient physiological responses and recovery dynamics, rather than as measures of ventilatory efficiency in the clinical sense.

Hormonal status also represents a potential source of variability. Although all participants reported regular menstrual cycles and no contraceptive use, testing was not synchronized with cycle phases, and fluctuations in estrogen and progesterone may have influenced oxygen kinetics, ventilatory responses, and fatigue.

Increasing sample size, implementing stricter control over recent training load, and standardizing hormonal and recovery-related factors would improve external validity and help determine whether the observed cardiopulmonary dynamics reflect volleyball-specific adaptations or transient functional limitations related to training status.

## 5. Conclusions

We observed a progressive decline in mechanical performance across successive CMJ repetitions, accompanied by increases in VO_2_, VCO_2_ and RER, indicating that a 4:1 recovery ratio did not allow cardiopulmonary variables to return to pre-exercise levels between bouts. While contact time remained relatively stable, suggesting a preserved movement pattern under increasing physiological strain, the persistence of elevated RER and VO_2_ points to a sustained cardiopulmonary and metabolic demand throughout the protocol. Within this cohort, the test therefore characterises how explosive neuromuscular output is maintained or lost under repeated efforts with incomplete recovery, rather than identifying specific mechanistic limitations at the muscular level. As such, the protocol may offer a practical framework for monitoring changes over time in the integrated mechanical and cardiopulmonary response to repeated jumping in competitive volleyball players, although larger studies are needed before its use for individual “readiness” decisions can be recommended.

## Figures and Tables

**Table 1:** Mechanical performance during the 0–5 s phase across repetitions 1–3.

		0–5 s			Statistical Results		
		Rep 1	Rep 2	Rep 3	*p* Value	*F* Value	*ηp* ^2^
**Mechanical Performance**	**Height**, cm	21.94 ± 3.00	23.03 ± 3.23	22.92 ± 3.79	0.084	2.687	0.151
	**Power**, w/kg	16.62 ± 2.48	17.06 ± 2.28	17.06 ± 2.71	0.450	0.820	0.051
	**Contact time**, s	0.69 ± 0.12	0.73 ± 0.16	0.68 ± 0.10	0.414	0.908	0.057

**Table 2:** Mechanical performance during the 5–10 s phase across repetitions 1–3.

		5–10 s			Statistical Results		
		Rep 1	Rep 2	Rep 3	*p* Value	*F* Value	*ηp* ^2^
**Mechanical Performance**	**Height**, cm	20.45 ± 2.73	20.38 ± 3.13	19.88 ± 3.20	0.214	1.621	0.097
	**Power**, w/kg	15.73 ± 2.60	15.51 ± 2.29	15.18 ± 2.25	0.201	1.690	0.101
	**Contact time**, s	0.72 ± 0.14	0.72 ± 0.12	0.73 ± 0.11	0.839	0.176	0.011

**Table 3:** Mechanical performance during the 10–15 s phase across repetitions 1–3.

		10–15 s			Statistical Results		
		Rep 1	Rep 2	Rep 3	*p* Value	*F* Value	*ηp* ^2^
**Mechanical Performance**	**Height**, cm	19.38 ± 2.99	18.32 ± 3.11	18.15 ± 3.19	0.006	6.100	0.289
	**Power**, w/kg	15.06 ± 2.67	14.20 ± 2.27	14.12 ± 2.20	0.012	5.044	0.251
	**Contact time**, s	0.73 ± 0.14	0.77 ± 0.17	0.75 ± 0.11	0.437	0.849	0.053

**Table 4:** CP during the 0–5 s phase across repetitions 1–3.

		0–5 s			Statistical Results		
		Rep 1	Rep 2	Rep 3	*p* Value	*F* Value	*ηp* ^2^
**CP parameters**	**VO_2_**, mL/min/kg	11.13 ± 4.74	17.55 ± 6.12	17.53 ± 2.80	0.0001	17.69	0.541
	**VO_2_/HR**, mL/b	6.51 ± 2.47	9.46 ± 3.09	8.94 ± 1.36	0.0008	9.15	0.378
	**VE/VO_2_**, L/min	20.32 ± 6.10	20.61 ± 4.79	34.59 ± 4.93	0.0001	56.41	0.789
	**VE/VCO_2_**, L/min	25.42 ± 7.23	26.10 ± 5.63	27.82 ± 2.89	0.2357	1.51	0.091
	**PetO_2_**, mmHg	107.9 ± 7.93	104.6 ± 6.54	114.1 ± 3.77	0.0297	3.96	0.209
	**PetCO_2_**, mmHg	29.47 ± 6.22	31.54 ± 5.52	36.71 ± 4.31	0.0006	9.57	0.389

**Table 5:** CP during the 5–10 s phase across repetitions 1–3.

		5–10 s			Statistical Results		
		Rep 1	Rep 2	Rep 3	*p* Value	*F* Value	*ηp* ^2^
**CP parameters**	**VO_2_**, mL/min/kg	17.55 ± 6.12	20 ± 5.81	22.65 ± 3.88	0.002	7.40	0.330
	**VO_2_/HR**, mL/b	9.46 ± 3.09	9.95 ± 2.85	10.77 ± 2.10	0.231	1.53	0.093
	**VE/VO_2_**, L/min	20.61 ± 4.79	31.71 ± 4.74	32.49 ± 4.78	0.0001	63.71	0.809
	**VE/VCO_2_**, L/min	26.10 ± 5.63	27.69 ± 2.68	27.89 ± 3.08	0.1789	1.82	0.108
	**PetO_2_**, mmHg	104.6 ± 6.54	112.4 ± 4.63	112 ± 5.42	0.0004	10.34	0.408
	**PetCO_2_**, mmHg	31.54 ± 5.52	35.75 ± 4.24	36.72 ± 4.34	0.0076	5.76	0.277

**Table 6:** CP during the 10–15 s phase across repetitions 1–3.

		10–15 s			Statistical Results		
		Rep 1	Rep 2	Rep 3	*p* Value	*F* Value	*ηp* ^2^
**CP parameters**	**VO_2_**, mL/min/kg	20.53 ± 5.70	24.23 ± 5.05	25.93 ± 4.71	0.0014	8.290	0.355
	**VO_2_/HR**, mL/b	10.13 ± 2.22	11.20 ± 2.40	11.53 ± 1.71	0.0552	3.194	0.175
	**VE/VO_2_**, L/min	23.36 ± 4.75	29.42 ± 4.45	30.68 ± 4.67	0.0001	35.04	0.700
	**VE/VCO_2_**, L/min	30.21 ± 6.37	28.04 ± 2.78	28.79 ± 3.20	0.1718	1.86	0.110
	**PetO_2_**, mmHg	103.1 ± 6.65	110.5 ± 5.17	111.3 ± 4.32	0.0001	22.70	0.602
	**PetCO_2_**, mmHg	32.19 ± 5.13	34.75 ± 4.23	34.45 ± 3.26	0.0160	4.75	0.240

## Data Availability

The original contributions presented in the study are included in the article, further inquiries can be directed to the corresponding author.

## Abbreviations

The following abbreviations are used in this manuscript:

CPET	Cardiopulmonary Exercise Testing
CMJ	Countermovement Jump
ECG	Electrocardiogram
HR	Heart rate
PETCO_2_	End-Tidal Carbon Dioxide Pressure
PETO_2_	End-Tidal Oxygen Pressure
RER	Respiratory Exchange Ratio
SD	Standard Deviation
VE	Minute Ventilation
VE/VCO_2_	Ventilatory Equivalent for Carbon Dioxide
VE/VO_2_	Ventilatory Equivalent for Oxygen
VCO_2_	Carbon Dioxide Output
VO_2_	Oxygen Uptake
VO_2_/HR	Oxygen Pulse (Oxygen Uptake per Heartbeat)
V1, V2, V3	Visit 1, Visit 2, Visit 3 (testing sessions)
